# The Telephone and Microphone in Auscultation

**Published:** 1880-12-20

**Authors:** C. J. Blake


### THE TELEPHONE AND MICROPHONE IN AUSCULTATION.

BY C. J. BLAKE, M.D.

It is too soon to have forgotten the enthusiasm which greeted the first publication of the electrical transmission of articulate sounds as made possible by the invention of the telephone, and the interest which was awakened in the medical profession in anticipation of its value in auscultation, and especially for the purposes of clinical demonstration—expectations which were still further encouraged at a later day by the results of experiments made almost simultaneously by Mr. Berliner, of Boston, and Prof. Eli W. Blake, of Brown University, in this country, and Prof. Hughes, in England, and published to the world by the latter in the various forms in which the discovery was demonstrable, as the microphone.

It is now more than four years since the introduction of the telephone, and more than two years since the appreciation of the full value of the "broken circuit" in connection with the telephone for sound transmission; in addition to the various forms of "transmitters" which are used on telephone lines, and which are in fact microphones, differing only in the mechanical devices for reception of the sound waves and adjustment of the contact surfaces, several forms of microphone have been constructed especially for purposes of auscultation, but none have as yet, even in a slight degree, answered this purpose. Setting careful investigation of their capabilities aside, this is sufficiently evidenced by the fact that none of these instruments have come into general use.

That, with numerous experimenters in the field, nothing in this line of research has been satisfactorily, practically accomplished awakens the question by those who await results as to why it has not been done, and it is an endeavor to answer this question which forms the basis of this paper, in which I shall have to apologize for partially retelling a twice-told tale in explanation of experiments bearing upon this subject made at different times during the past four years, at first with the hope of devising an auscultation telephone and microphone, and finally for the purpose of determining the reasons for the want of success in this attempt.

Having failed at one end, it was necessary to begin afresh at the other; this I am inclined to think has been the experience of other investigators of this subject, and as achievement is so often, finally, the result of the study of apparent impossibilities, it is to be hoped that a practically useful auscultation microphone may yet be devised. When the discoveries of the telephone and microphone were first announced, some very enthusiastic gentlemen went so far as to predict that telephonic consultations would be held, and that eminent special practitioners, who "listened to the heart-beats of a nation," as a matter of business, would each settle themselves down in the centre of a web of wires and auscult at indefinite distances from the patients; the general argument being that an instrument which could make audible the footfall of a fly or the rustling of a camel's-hair pencil could certainly transmit at their full value the sounds from within the chest cavity which are so loudly heard in the stethoscope. But little of that knowledge of the practical working of the telephone over the lines in use for purposes of ordinary communication, which came with the general introduction of the instrument, was necessary to prove the baselessness of this scheme; the very delicacy of the telephone in its susceptibility to interrupted currents and its almost fatal propensity—if such an expression may be used—to pick up sounds that did not belong to it were enough to show that it could not be used for so delicate a purpose as auscultation over any circuit of sufficient length to expose it to the influence of other electric currents. The first experiments, begun in 1877, were therefore made over a private wire, extending a distance of about eight hundred feet from one house to another, overhead, isolated as far as possible from other wires, the nearest overhead wire being six feet distant, but having a ground connection; the telephones used were the ordinary hard-rubber case-hand telephones, Bell. The telephone was placed upon the bared surface of the chest, the mouth-piece of the telephone being pressed upon the surface, the auscultant listening at the second telephone at the other end of the line. The experiment was several times repeated with the telephone upon different portions of the chest, and with varying degrees of pressure; with exception, in one instance only, of the suspicion of a barely perceptible "thud," no sound which could be referred to the heart as its source was heard, although with the telephone in this position the voice and words of the experimenter could be heard by the auscultant.

There could also be plainly heard, in consequence of the ground connection, the snapping and crackling noises indicative of earth currents, the clicking of the Morse instruments, and the sound of a "fast speed transmitter" on the Western Union lines running along the Providence railroad, and the ticking of the clock connected with the Observatory in Cambridge. It was very plain, therefore, that even if the heart-sounds could be transmitted they would be drowned by extraneous noises, and the experiment was repeated by making the auscultation in the same room with the patient, the two telephones being connected by the short flexible wires, about three feet long, in common use. Even under these favorable circumstances no sound originating in the chest cavity could be heard.

Disks of postal-card paper, having small disks of iron in the centre, were substituted for the metal disks of both telephones, but with no better results, and the experiments with the telephone alone were abandoned.

The introduction of the microphone awakened new interest in the possibilities of auscultation, and a series of experiments was instituted with various forms of this instrument, the Bell telephone being used as a receiver.

Guided by former experience, a short line without ground connections was always used, and the microphone was placed either directly upon the chest or upon a small box of proportions suitable to its action as a resonator for very low tones. In no instance were sounds heard in the telephone which could be referred to the heart or lungs as their source, but there could be distinctly heard rubbing and rustling noises resulting from the contact of the microphone apparatus with the surface of the skin and the small hairs upon it; and when the microphone was held in the hand, a short distance above the surface of the chest, noises could be heard which could be referred only to the disturbance of the opposing microphonic surfaces in consequence of the slight involuntary movements of the hand. Auscultation of the tracheal respiration was also attempted, with the same results. Of the various forms of microphone used in these experiments one was the transmitter invented by Mr. Francis Blake and used by the National Bell Telephone Company; another a microphone having a carved membrane, modeled upon the human membrana tympani; and a third a common form of microphone, but arranged with an adjustment which seemed to fit it particularly for the transmission of low tones. In order to test the capactty of the latter instrument in this respect, a rasonator was turned as near as possible to the pitch of the first beat of the heart (in this case a tone of about 170 v. s.) and placed upon the microphone. On blowing into the resonator forcibly, and thereby giving a tone much louder than that of the heart, there could be heard in the telephone a rushing sound of comparatively high pitch, but no fundamental tone of the resonator.

A large tuning-fork of the same pitch, set in vibration and held over the microphone, or placed upon the table at a short distance from it, was distinctly heard.

It seemed, therefore, that the microphone would not transmit audibly tones as low in pitch as those of the heart-beats unless they were of considerable intensity; in other words, the volume of sound from the heart would need to be greatly increased, or its pitch considerably raised, in order to make it audible by microphonic auscultation with any present apparatus. The susceptibility of the microphone, moreover, to movements causing slight disturbances of its contact surfaces and the correspondingly loud noises induced in the telephone present a very serious difficulty in the way of auscultation.

In other words, for successful auscultation a microphone must be constructed which will respond only to very low tones of slight intensity, the contact surfaces of which shall not be subject to slight mechanical disturbance, and the adjustment of which shall be fairly permanent. In view of the work in electrical sound transmission during the past four years, no one would be so rash as to assert that these conditions cannot be fulfilled.

The reasons for the failure in auscultation as above stated, are:

(1.) In the telephones, the very considerable loss of power in sound transmission. In a series of experiments made for another purpose in 1878 it was found that in response to a tone of 448 v. s. the centre of the disk of the transmitting telephone, without the magnet, moved through a space of 0.2625 millimetre, while the disk of the receiving telephone made a corresponding movement of only 0.0135 millimetre, a loss in motion of 92.9 per cent. between the two telephone disks. With so great a loss by telephonic transmission in a tone of considerable intensity, it is claimed that the proportion of so weak and dull a sound as that of the heart, its overtones damped by transmission through soft tissues, given forth by the receiving telephone, would be inaudible to the human ear.

(2.) While the telephone creates its own current of electricity, a certain proportion of the working force which moves the disk of the transmitting instrument being expended in this operation, the microphone takes a current already provided by a battery, and merely varies the amount of the current passing from one to the other of its opposing contact surfaces. The whole of the motive force, therefore, generally speaking, is expended in varying the resistance. With this great saving the instrument is much more susceptible to very slight sounds, but it is also fatally susceptible to the influenee of very slight mechanical movements which vary the relations of the contact surfaces to each other, thereby varying the amount of current passing, and producing corresponding sounds in the telephone. These sounds are usually very loud and sharp, and interfere materially with the hearing as well as with the transmission of regular musical tones. It may be briefly put that for the delicate purpose in question the telephone transmits too little, and the microphone, in any of its present forms, transmits too much.

Several auscultation microphones and sphygmophones have been constructed in France and Germany. I have had no opportunity to experiment with them, but from the details of their construction they do not differ essentially from those used in these experiments, and I should judge them to be open to the same objections.—*Boston Med. and Surg. Journal.*

